# Supplementary material for: Improved cartilage regeneration by implantation of acellular biomaterials after bone marrow stimulation: a systematic review and meta-analysis of animal studies
Source: PeerJ. 2016 Sep 8;4:e2243. doi: 10.7717/peerj.2243 (PMC5018675; doi:10.7717/peerj.2243)

Supplementary Information 2. Methodological quality assessment: questions and scoring flowchart.

Questions

1) Randomization: Was the allocation sequence adequately generated and applied (selection bias)?

2) Baseline characteristics: Were the groups similar at baseline (selection bias)?

Were all groups implanted in one animal and where there differences in load bearing between implantation sites (2.1), and if not all groups were in one animal, was randomization adequately performed?

How were animal characteristics divided over the groups (2.2)?

Where there differences between groups at the moment of surgical intervention (2.3)?

3) Implantation concealment: Was the allocation adequately concealed (selection bias)?

4) Random housing: Were the animals randomly housed during the experiment (performance bias)?

5) Blinding: Where the caregivers and/or investigators blinded from knowledge which intervention each animal received during the experiments (performance bias)?

6) Random outcome assessment: Were the animals selected at random for outcome assessment (detection bias)?

7) Analysis blinding: Was the outcome assessor blinded (detection bias)?

8) Incomplete outcome data: Were incomplete outcome data adequately addressed (attrition bias)?

Flowchart

1) Was the allocation sequence adequately generated and applied?

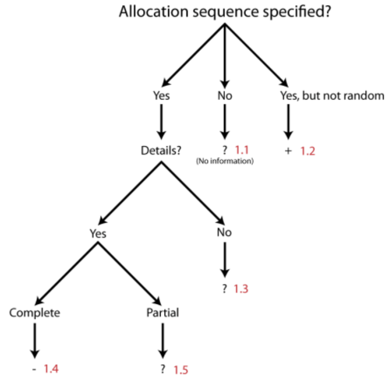

2) Were the groups similar at baseline or were they adjusted for confounders in the analysis

Part 1: # conditions/animal?

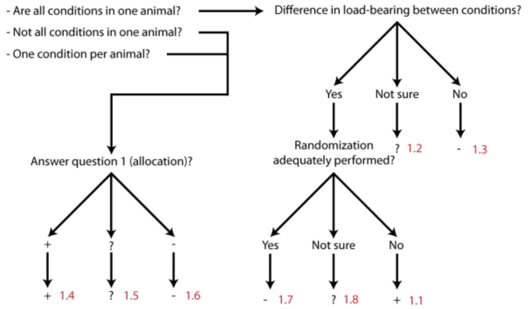

Part 2: Dividing of charactertics over groups (sex, age, weight)?

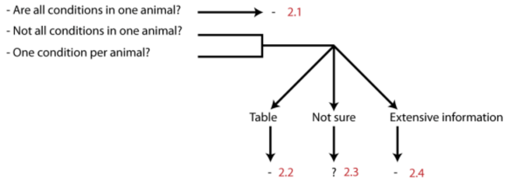

Part 3: Difference between groups at moment of surgical intervention?

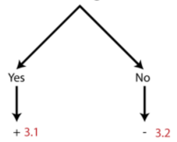

3) Was the allocation adequately concealed?

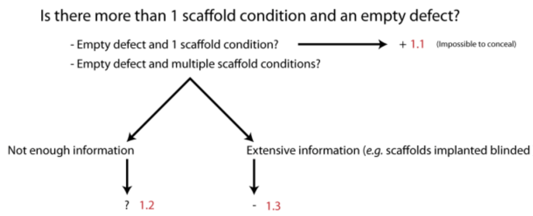

4) Were the animals randomly housed during the experiment?

5) Were the caregivers and/or investigators blinded from knowledge which intervention each animal received during the experiment?

6) Were the animals selected at random for outcome assessment?

In general, for this type of *in vivo* studies these domains are not essential to be assessed. Therefore, only specific examples were reported in footnotes.

7) Was the outcome assessor blinded?

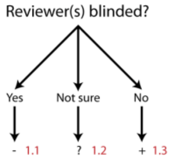

8) Were incomplete outcome data adequatly addressed?

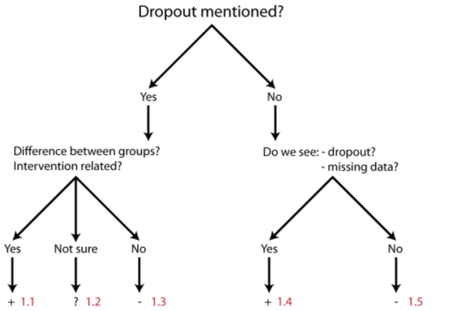

Supplement: Supplemental Information 2 [file peerj-04-2243-s002.pdf]
