# Supplementary material for: Improved cartilage regeneration by implantation of acellular biomaterials after bone marrow stimulation: a systematic review and meta-analysis of animal studies
Source: PeerJ. 2016 Sep 8;4:e2243. doi: 10.7717/peerj.2243 (PMC5018675; doi:10.7717/peerj.2243)
Supplement: Supplemental Information 4 [file peerj-04-2243-s004.pdf]

Supplementary Information 4. Study characteristics of included studies: abbreviations and references

Abbreviations

Biomaterial

ACI: autologous chondrocyte implantation
BCP: bicalcium phosphate
CDBM: cancellous demineralized bone matrix
CS: chondroitin sulfate
DABM: demineralized allogeneic bone matrix
DCBM: decellularized cancellous bone matrix
DCCBM: decalcified cortical-cancellous bone matrix
DTBM: demineralized trabecular bone matrix
DCBM: demineralized cortical bone matrix
DCM: decellularized cartilage matrix
GMP: gelatin microspheres
ICP: Injectable calcium phosphate
KLD hydrogel: self-assembling hydrogel from peptide sequences $(RADA)_4$ and $(KLDL)_3$
LLDPE: low-density polyethylene
MA: methacrylate
OPF: oligo(poly(ethylene glycol) fumarate)
PAMPS-PDMAAm DN hydrogel: poly-(2-Acrylamido-2-methylpropane sulfonic acid/poly-(N,N'-Dimetyl acrylamide double network hydrogel
PCL: polycaprolactone
PCLPU: polycaprolactone-polyurethane
PEG: poly(ethylene glycol)
PEOT/PBT: poly(ethylene oxide terephthalate)/poly(butylene terephthalate)
PHBV: (poly(3-hydroxybutyric acid-co-3-hydroxyvaleric acid)
pHEMA-[MMA-co-AA]: poly(2-hydroxyethyl methacrylate)-poly(methacrylate of acrylic methyl co-acid
PGA: poly(glycolic acid)
PLA: poly(lactic acid)
PLCL: poly(L-lactide-co-caprolactone)
PLGA: poly(lactic-co-glycolic acid)
PDLA: poly(d-lactide)
PVA: poly(vinyl alcohol)
SPU: segmented polyurethane
TCP: tricalcium phosphate
TMC: N,N,N-trimethylchitosanchloride
UHMWPE: Ultra-high molecular weight polyethylene

Cells

ACI: autologous chondrocyte implantation
ASCs: adipose-derived stem cells
BM-MNCs: bone marrow-derived mononuclear cells
EPCs: endothelial progenitor cells
MPCs: bone marrow-derived progenitor cells
MSCs: mesenchymal stem cells
SDMSCs: synovium derived mesenchymal stem cells
BMAC: bone marrow aspiration concentrate

Biologicals

BMP: bone morphogenetic protein
bBMPs: bovine bone morphogenetic proteins
FGF: fibroblast growth factor

IGF: insulin-like growth factor
OP-1: osteogenic protein-1 (BMP-7)
PRP: platelet-rich plasma
SDF-1: stromal cell-derived factor-1
SVF: stromal vascular fraction
TGF-β: transforming growth factor-β

Conditions
IAM: intermittent active motion
CPM: continuous passive motion
Imm: immobilized

References

1 Abarrategi A, Lopiz-Morales Y, Ramos V, Civantos A, Lopez-Duran L, Marco F, and Lopez-Lacomba JL. 2010. Chitosan scaffolds for osteochondral tissue regeneration. *Journal of Biomedical Materials Research Part A* 95:1132-1141. 10.1002/jbm.a.32912

2 Ahn JH, Lee TH, Oh JS, Kim SY, Kim HJ, Park IK, Choi BS, and Im GI. 2009. Novel hyaluronate-atelocollagen/beta-TCP-hydroxyapatite biphasic scaffold for the repair of osteochondral defects in rabbits. *Tissue Engineering Part A* 15:2595-2604. 10.1089/ten.TEA.2008.0511

3 Albrecht F, Roessner A, and Zimmermann E. 1983. Closure of osteochondral lesions using chrondral fragments and fibrin adhesive. *Archives of Orthopaedic and Trauma Surgery* 101:213-217.

4 Arakaki K, Kitamura N, Fujiki H, Kurokawa T, Iwamoto M, Ueno M, Kanaya F, Osada Y, Gong JP, and Yasuda K. 2010. Artificial cartilage made from a novel double-network hydrogel: In vivo effects on the normal cartilage and ex vivo evaluation of the friction property. *Journal of Biomedical Materials Research Part A* 93:1160-1168. 10.1002/jbm.a.32613

5 Arakaki K, Kitamura N, Kurokawa T, Onodera S, Kanaya F, Gong JP, and Yasuda K. 2011. Joint immobilization inhibits spontaneous hyaline cartilage regeneration induced by a novel double-network gel implantation. *Journal of Materials Science: Materials in Medicine* 22:417-425. 10.1007/s10856-010-4216-0

6 Araki S, Imai S, Ishigaki H, Mimura T, Nishizawa K, Ueba H, Kumagai K, Kubo M, Mori K, Ogasawara K, and Matsusue Y. 2015. Improved quality of cartilage repair by bone marrow mesenchymal stem cells for treatment of an osteochondral defect in a cynomolgus macaque model. *Acta Orthopaedica* 86:119-126. 10.3109/17453674.2014.958807

7 Athanasiou K, Korvick D, and Schenck Jr R. 1997. Biodegradable implants for the treatment of osteochondral defects in a goat model. *Tissue Engineering* 3:363-373. 10.1089/ten.1997.3.363

8 Aulin C, Jensen-Waern M, Ekman S, Hagglund M, Engstrand T, Hilborn J, and Hedenqvist P. 2013. Cartilage repair of experimentally 11 induced osteochondral defects in new zealand white rabbits. *Laboratory Animals* 47:58-65. 10.1177/0023677212473716

9 Bai T, Shu J, Wang J, Lu J, Li W, and Pu B. 2008. Experimental research on repair of rabbit articular cartilage deffects with composite of autologous cell-carriers. *Chinese Journal of Reparative and Reconstructive Surgery* 22:487-491.

10 Bal BS, Rahaman MN, Jayabalan P, Kuroki K, Cockrell MK, Yao JQ, and Cook JL. 2010. In vivo outcomes of tissue-engineered osteochondral grafts. *Journal of Biomedical Materials Research Part B* 93:164-174. 10.1002/jbm.b.31571

11 Barron V, Merghani K, Shaw G, Coleman CM, Hayes JS, Ansboro S, Manian A, O'Malley G, Connolly E, Nandakumar A, van Blitterswijk CA, Habibovic P, Moroni L, Shannon F, Murphy JM, and Barry F. 2015. Evaluation of Cartilage Repair by Mesenchymal Stem Cells Seeded on a PEOT/PBT Scaffold in an Osteochondral Defect. *Annals of Biomedical Engineering*. 10.1007/s10439-015-1246-2

12 Bavaresco VP, Garrido L, Batista NA, Malmonge SM, and Belangero WD. 2008. Mechanical and morphological evaluation of osteochondral implants in dogs. *Artificial Organs* 32:310-316. 10.1111/j.1525-1594.2008.00548.x

13 Bell AD, Lascau-Coman V, Sun J, Chen G, Lowerison MW, Hurtig MB, and Hoemann CD. 2013. Bone-Induced Chondroinduction in Sheep Jamshidi Biopsy Defects with and without Treatment by Subchondral Chitosan-Blood Implant: 1-Day, 3-Week, and 3-Month Repair. *Cartilage* 4:131-143. 1177/1947603512463227

14 Betsch M, Schneppendahl J, Thuns S, Herten M, Sager M, Jungbluth P, Hakimi M, and Wild M. 2013. Bone Marrow Aspiration Concentrate and Platelet Rich Plasma for Osteochondral Repair in a Porcine Osteochondral Defect Model. *PLoS One* 8. 10.1371/journal.pone.0071602

15 Betsch M, Thelen S, Santak L, Herten M, Jungbluth P, Miersch D, Hakimi M, and Wild M. 2014. The role of erythropoietin and bone marrow concentrate in the treatment of osteochondral defects in mini-pigs. *PLoS One* 9:e92766. 10.1371/journal.pone.0092766

16 Bichara DA, Bodugoz-Sentruk H, Ling D, Malchau E, Bragdon CR, and Muratoglu OK. 2014. Osteochondral defect repair using a polyvinyl alcohol-polyacrylic acid (PVA-PAAc) hydrogel. *Biomedical Materials* 9. 10.1088/1748-6041/9/4/045012

17 Breinan HA, Martin SD, Hsu HP, and Spector M. 2000. Healing of canine articular cartilage defects treated with microfracture, a type-II collagen matrix, or cultured autologous chondrocytes. *Journal of Orthopaedic Research* 18:781-789. 10.1002/jor.1100180516

18 Brittberg M, Sjogren-Jansson E, Lindahl A, and Peterson L. 1997. Influence of fibrin sealant (Tisseel) on osteochondral defect repair in the rabbit knee. *Biomaterials* 18:235-242. 10.1016/S0142-9612(96)00117-2

19 Buma P, Pieper JS, van Tienen T, van Susante JL, van der Kraan PM, Veerkamp JH, van den Berg WB, Veth RP, and van Kuppevelt TH. 2003. Cross-linked type I and type II collagenous matrices for the repair of full-thickness articular cartilage defects--a study in rabbits. *Biomaterials* 24:3255-3263.

20 Caminal M, Peris D, Fonseca C, Barrachina J, Codina D, Rabanal RM, Moll X, Morist A, Garcia F, Cairo JJ, Godia F, Pla A, and Vives J. 2015. Cartilage resurfacing potential of PLGA scaffolds loaded with autologous cells from cartilage, fat, and bone marrow in an ovine model of osteochondral focal defect. *Cytotechnology*. 10.1007/s10616-015-9842-4

21 Cao Z, Hou S, Sun D, Wang X, and Tang J. 2012. Osteochondral regeneration by a bilayered construct in a cell-free or cell-based approach. *Biotechnology Letters* 34:1151-1157. 10.1007/s10529-012-0884-9

22 Chang CH, Kuo TF, Lin FH, Wang JH, Hsu YM, Huang HT, Loo ST, Fang HW, Liu HC, and Wang WC. 2011. Tissue engineering-based cartilage repair with mesenchymal stem cells in a porcine model. *Journal of Orthopaedic Research* 29:1874-1880. 10.1002/jor.21461

23 Chang NJ, Lam CF, Lin CC, Chen WL, Li CF, Lin YT, and Yeh ML. 2013a. Transplantation of autologous endothelial progenitor cells in porous PLGA scaffolds create a microenvironment for the regeneration of hyaline cartilage in rabbits. *Osteoarthritis Cartilage* 21:1613-1622. 10.1016/j.joca.2013.07.016

24 Chang NJ, Lin CC, Li CF, Su K, and Yeh ML. 2013b. The effect of osteochondral regeneration using polymer constructs and continuous passive motion therapy in the lower weight-bearing zone of femoral trocheal groove in rabbits. *Annals of Biomedical Engineering* 41:385-397. 10.1007/s10439-012-0656-7

25 Chang NJ, Lin CC, Li CF, Wang DA, Issariyaku N, and Yeh ML. 2012. The combined effects of continuous passive motion treatment and acellular PLGA implants on osteochondral regeneration in the rabbit. *Biomaterials* 33:3153-3163. 10.1016/j.biomaterials.2011.12.054

26 Chen H, Yang X, Liao Y, Zeng X, Liang P, Kang N, Tan J, and Liang Z. 2011a. MRI and histologic analysis of collagen type II sponge on repairing the cartilage defects of rabbit knee joints. *Journal of Biomedical Materials Research Part B* 96 B:267-275. 10.1002/jbm.b.31762

27 Chen J, Chen H, Li P, Diao H, Zhu S, Dong L, Wang R, Guo T, Zhao J, and Zhang J. 2011b. Simultaneous regeneration of articular cartilage and subchondral bone in vivo using MSCs

induced by a spatially controlled gene delivery system in bilayered integrated scaffolds. *Biomaterials* 32:4793-4805. 10.1016/j.biomaterials.2011.03.041
28 Chen P, Tao J, Zhu S, Cai Y, Mao Q, Yu D, Dai J, and Ouyang H. 2015. Radially oriented collagen scaffold with SDF-1 promotes osteochondral repair by facilitating cell homing. *Biomaterials* 39:114-123. 10.1016/j.biomaterials.2014.10.049
29 Chen WC, Yao CL, Wei YH, and Chu IM. 2011c. Evaluating osteochondral defect repair potential of autologous rabbit bone marrow cells on type II collagen scaffold. *Cytotechnology* 63:13-23. 10.1007/s10616-010-9314-9
30 Chen Z, Zhao M, Liu K, Wan Y, Li X, and Feng G. 2014. Novel chitosan hydrogel formed by ethylene glycol chitosan, 1,6-diisocyanatohexan and polyethylene glycol-400 for tissue engineering scaffold: in vitro and in vivo evaluation. *Journal of Materials Science: Materials in Medicine* 25:1903-1913. 10.1007/s10856-014-5223-3
31 Cheng A, Kapacee Z, Peng J, Lu S, Lucas RJ, Hardingham TE, and Kimber SJ. 2014. Cartilage repair using human embryonic stem cell-derived chondroprogenitors. *Stem Cells Translational Medicine* 3:1287-1294. 10.5966/sctm.2014-0101
32 Chevrier A, Hoemann CD, Sun J, and Buschmann MD. 2007. Chitosan-glycerol phosphate/blood implants increase cell recruitment, transient vascularization and subchondral bone remodeling in drilled cartilage defects. *Osteoarthritis Cartilage* 15:316-327. 10.1016/j.joca.2006.08.007
33 Chevrier A, Hoemann CD, Sun J, and Buschmann MD. 2011. Temporal and spatial modulation of chondrogenic foci in subchondral microdrill holes by chitosan-glycerol phosphate/blood implants. *Osteoarthritis and Cartilage* 19:136-144. 10.1016/j.joca.2010.10.026
34 Christensen BB, Foldager CB, Hansen OM, Kristiansen AA, Le DQ, Nielsen AD, Nygaard JV, Bunger CE, and Lind M. 2012. A novel nano-structured porous polycaprolactone scaffold improves hyaline cartilage repair in a rabbit model compared to a collagen type I/III scaffold: in vitro and in vivo studies. *Knee Surgery, Sports Traumatology, Arthroscopy* 20:1192-1204. 10.1007/s00167-011-1692-9
35 Chung JY, Song M, Ha CW, Kim JA, Lee CH, and Park YB. 2014. Comparison of articular cartilage repair with different hydrogel-human umbilical cord blood-derived mesenchymal stem cell composites in a rat model. *Stem Cells Translational Medicine* 5. 10.5966/sctm.2014-0264
36 Coburn JM, Gibson M, Monagle S, Patterson Z, and Elisseeff JH. 2012. Bioinspired nanofibers support chondrogenesis for articular cartilage repair. *Proceedings of the National Academy of Sciences* 109:10012-10017. 10.1073/pnas.1121605109
37 Cohen SB, Meirisch CM, Wilson HA, and Diduch DR. 2003. The use of absorbable co-polymer pads with alginate and cells for articular cartilage repair in rabbits. *Biomaterials* 24:2653-2660. 10.1016/S0142-9612(03)00058-9
38 Cui Y, Wu J, and Hu Y. 2007. Repair of articular cartilage defect with poly-lactide-co-glycolide loaded with recombinant human bone morphogenetic protein in rabbits. *Chinese Journal of Reparative and Reconstructive Surgery* 21:1233-1237.
39 Cui Y, Wu J, and Hu Y. 2008. The comparative study on the reparative effect of PLGA and collagen sponge combined with BMP on the articular cartilage defect of rabbits. *Chinese Journal of Reparative and Reconstructive Surgery* 22:148-152.
40 Cui YM, Wu J, and Hu YY. 2009. Repairing articular cartilage defects in rabbits using bone marrow stromal cell-derived chondrocytes compounded with poly(lactic-co-glycolic acid). *Journal of Clinical Rehabilitative Tissue Engineering Research* 13:10049-10054. 10.3969/j.issn.1673-8225.2009.51.009
41 Cui Z, Wright LD, Guzzo R, Freeman JW, Drissi H, and Nair LS. 2013. Poly(d-lactide)/poly(caprolactone) nanofiber-thermogelling chitosan gel composite scaffolds for osteochondral tissue regeneration in a rat model. *Journal of Bioactive and Compatible Polymers* 28:115-125. 10.1177/0883911512472278

42 Dahlberg L, and Kreicbergs A. 1991. Demineralized allogeneic bone matrix for cartilage repair. *Journal of Orthopaedic Research* 9:11-19. 10.1002/jor.1100090103

43 Dahlin RL, Kinard LA, Lam J, Needham CJ, Lu S, Kasper FK, and Mikos AG. 2014. Articular chondrocytes and mesenchymal stem cells seeded on biodegradable scaffolds for the repair of cartilage in a rat osteochondral defect model. *Biomaterials* 35:7460-7469. 10.1016/j.biomaterials.2014.05.055

44 Dai L, He Z, Zhang X, Hu X, Yuan L, Qiang M, Zhu J, Shao Z, Zhou C, and Ao Y. 2014. One-step repair for cartilage defects in a rabbit model: a technique combining the perforated decalcified cortical-cancellous bone matrix scaffold with microfracture. *The American Journal of Sports Medicine* 42:583-591. 10.1177/0363546513518415

45 de Mulder EL, Hannink G, van Kuppevelt TH, Daamen WF, and Buma P. 2014. Similar hyaline-like cartilage repair of osteochondral defects in rabbits using isotropic and anisotropic collagen scaffolds. *Tissue Engineering Part A* 20:635-645. 10.1089/ten.TEA.2013.0083

46 Deng T, Lv J, Pang J, Liu B, and Ke J. 2012. Construction of tissue-engineered osteochondral composites and repair of large joint defects in rabbit. *Journal of Tissue Engineering and Regenerative Medicine*. 10.1002/term.1556

47 Di W, Zhao ZJ, Zhu XS, Hao Y, Qing L, and Ke M. 2010. Calcium alginate beads combined with autologous chondrocyte for repair of adult rabbit articular cartilage injury. *Journal of Clinical Rehabilitative Tissue Engineering Research* 14:3811-3814.

48 Diduch DR, Jordan LC, Mierisch CM, and Balian G. 2000. Marrow stromal cells embedded in alginate for repair of osteochondral defects. *Arthroscopy* 16:571-577. 10.1053/jars.2000.4827

49 Ding XP, Jin XQ, Wang ZY, and Fu YL. 2008. Repairing articular cartilage defects in rabbits by gene-modified stem cells transplantation of biodegradable materials. *Journal of Clinical Rehabilitative Tissue Engineering Research* 12:1839-1842.

50 Dorotka R, Bindreiter U, Macfelda K, Windberger U, and Nehrer S. 2005. Marrow stimulation and chondrocyte transplantation using a collagen matrix for cartilage repair. *Osteoarthritis Cartilage* 13:655-664. 10.1016/j.joca.2005.04.001

51 Dresing I, Zeiter S, Auer J, Alini M, and Eglin D. 2014. Evaluation of a press-fit osteochondral poly(ester-urethane) scaffold in a rabbit defect model. *Journal of Materials Science: Materials in Medicine*. 10.1007/s10856-014-5192-6

52 Duan P, Pan Z, Cao L, He Y, Wang H, Qu Z, Dong J, and Ding J. 2014. The effects of pore size in bilayered poly(lactide-co-glycolide) scaffolds on restoring osteochondral defects in rabbits. *Journal of Biomedical Materials Research Part A* 102:180-192. 10.1002/jbm.a.34683

53 Duan X, Zhu X, Dong X, Yang J, Huang F, Cen S, Leung F, Fan H, and Xiang Z. 2013. Repair of large osteochondral defects in a beagle model with a novel type I collagen/glycosaminoglycan-porous titanium biphasic scaffold. *Materials Science and Engineering: C* 33:3951-3957. 10.1016/j.msec.2013.05.040

54 Endo J, Watanabe A, Sasho T, Yamaguchi S, Saito M, Akagi R, Muramatsu Y, Mukoyama S, Katsuragi J, Akatsu Y, Fukawa T, Okubo T, Osone F, and Takahashi K. 2015. Utility of T2 mapping and dGEMRIC for evaluation of cartilage repair after allograft chondrocyte implantation in a rabbit model. *Osteoarthritis Cartilage* 23:280-288. 10.1016/j.joca.2014.10.012

55 Enea D, Guerra D, Roggiani J, Cecconi S, Manzotti S, Quaglino D, Pasquali-Ronchetti I, and Gigante A. 2013. Mixed type I and type II collagen scaffold for cartilage repair: Ultrastructural study of synovial membrane response and healing potential versus microfractures (a pilot study). *International Journal of Immunopathology and Pharmacology* 26:917-930. 10.1088/2058-7058/26/06/10

56 Erggelet C, Endres M, Neumann K, Morawietz L, Ringe J, Haberstroh K, Sittinger M, and Kaps C. 2009. Formation of cartilage repair tissue in articular cartilage defects pretreated with microfracture and covered with cell-free polymer-based implants. *Journal of Orthopaedic Research* 27:1353-1360. 10.1002/jor.20879

57 Erggelet C, Neumann K, Endres M, Haberstroh K, Sittinger M, and Kaps C. 2007. Regeneration of ovine articular cartilage defects by cell-free polymer-based implants. *Biomaterials* 28:5570-5580. 10.1016/j.biomaterials.2007.09.005

58 Feczko P, Hangody L, Varga J, Bartha L, Dioszegi Z, Bodo G, Kendik Z, and Modis L. 2003. Experimental results of donor site filling for autologous osteochondral mosaicplasty. *Arthroscopy* 19:755-761. 10.1016/S0749-8063(03)00402-X

59 Fernandez FB, Shenoy S, Suresh Babu S, Varma HK, and John A. 2012. Short-term studies using ceramic scaffolds in lapine model for osteochondral defect amelioration. *Biomedical Materials* 7. 10.1088/1748-6041/7/3/035005

60 Ferretti M, Marra KG, Kobayashi K, Defail AJ, and Chu CR. 2006. Controlled in vivo degradation of genipin crosslinked polyethylene glycol hydrogels within osteochondral defects. *Tissue Engineering* 12:2657-2663. 10.1089/ten.2006.12.2657

61 Filova E, Jelinek F, Handl M, Lytvynets A, Rampichova M, Varga F, Cinatl J, Soukup T, Trc T, and Amler E. 2008. Novel composite hyaluronan/type I collagen/fibrin scaffold enhances repair of osteochondral defect in rabbit knee. *Journal of Biomedical Materials Research Part B* 87:415-424. 10.1002/jbm.b.31119

62 Filova E, Rampichova M, Handl M, Lytvynets A, Halouzka R, Usvald D, Hlucilova J, Prochazka R, Dezortova M, Rolencova E, Kostakova E, Trc T, Stastny E, Kolacna L, Hajek M, Motlik J, and Amler E. 2007. Composite hyaluronate-type I collagen-fibrin scaffold in the therapy of osteochondral defects in miniature pigs. *Physiological Research* 56:S5-S16.

63 Filova E, Rampichova M, Litvinec A, Drzik M, Mickova A, Buzgo M, Kostakova E, Martinova L, Usvald D, Prosecka E, Uhlik J, Motlik J, Vajner L, and Amler E. 2013. A cell-free nanofiber composite scaffold regenerated osteochondral defects in miniature pigs. *International Journal of Pharmaceutics* 447:139-149. 10.1016/j.ijpharm.2013.02.056

64 Fonseca C, Caminal M, Peris D, Barrachina J, Fabregas PJ, Garcia F, Cairo JJ, Godia F, Pla A, and Vives J. 2014. An arthroscopic approach for the treatment of osteochondral focal defects with cell-free and cell-loaded PLGA scaffolds in sheep. *Cytotechnology* 66:345-354. 10.1007/s10616-013-9581-3

65 Fragonas E, Valente M, Pozzi-Mucelli M, Toffanin R, Rizzo R, Silvestri F, and Vittur F. 2000. Articular cartilage repair in rabbits by using suspensions of allogenic chondrocytes in alginate. *Biomaterials* 21:795-801. 10.1016/S0142-9612(99)00241-0

66 Frenkel SR, Bradica G, Brekke JH, Goldman SM, Ieska K, Issack P, Bong MR, Tian H, Gokhale J, Coutts RD, and Kronengold RT. 2005. Regeneration of articular cartilage--evaluation of osteochondral defect repair in the rabbit using multiphasic implants. *Osteoarthritis Cartilage* 13:798-807. 10.1016/j.joca.2005.04.018

67 Frenkel SR, Toolan B, Menche D, Pitman MI, and Pachence JM. 1997. Chondrocyte transplantation using a collagen bilayer matrix for cartilage repair. *The Journal of Bone and Joint Surgery. British Volume* 79:831-836.

68 Fujimoto E, Ochi M, Kato Y, Mochizuki Y, Sumen Y, and Ikuta Y. 1999. Beneficial effect of basic fibroblast growth factor on the repair of full-thickness defects in rabbit articular cartilage. *Archives of Orthopaedic and Trauma Surgery* 119:139-145.

69 Fukuda A, Kato K, Hasegawa M, Hirata H, Sudo A, Okazaki K, Tsuta K, Shikinami Y, and Uchida A. 2005. Enhanced repair of large osteochondral defects using a combination of artificial cartilage and basic fibroblast growth factor. *Biomaterials* 26:4301-4308. 10.1016/j.biomaterials.2004.11.007

70 Fukui T, Kitamura N, Kurokawa T, Yokota M, Kondo E, Gong JP, and Yasuda K. 2014. Intra-articular administration of hyaluronic acid increases the volume of the hyaline cartilage regenerated in a large osteochondral defect by implantation of a double-network gel. *Journal of Materials Science: Materials in Medicine* 25:1173-1182. 10.1007/s10856-013-5139-3

71 Gang J, Wei-yuan X, Ya Z, Xing-xiang Z, Fei Y, and Wei-ping S. 2011. Repair of rabbit articular cartilage and subchondral defects using porous silk fibroin/hydroxyapatite combined with

adipose-derived stromal cells. *Journal of Clinical Rehabilitative Tissue Engineering Research* 15:5327-5333.

72 Gao J, Dennis JE, Solchaga LA, Goldberg VM, and Caplan AI. 2002. Repair of osteochondral defect with tissue-engineered two-phase composite material of injectable calcium phosphate and hyaluronan sponge. *Tissue Engineering Part A* 8:827-837. 10.1089/10763270260424187

73 Gao J, Knaack D, Goldberg VM, and Caplan AI. 2004. Osteochondral defect repair by demineralized cortical bone matrix. *Clinical Orthopaedics and Related Research* 427S:S62-S66.

74 Gao SJ, Wei JC, Lu B, Shao DC, Li T, Chen JQ, and Wang JZ. 2012. Experimental research on repairing full-thickness articular cartilage defects by transplantation of autologous uncultured bone-marrow-derived mononuclear cells in combination with micro-fracture. *Chinese Medical Journal* 92:2463-2467. 10.3760/cma.j.issn.0376-2491.2012.35.005

75 Getgood A, Henson F, Skelton C, Herrera E, Brooks R, Fortier LA, and Rushton N. 2012a. The Augmentation of a Collagen/Glycosaminoglycan Biphasic Osteochondral Scaffold with Platelet-Rich Plasma and Concentrated Bone Marrow Aspirate for Osteochondral Defect Repair in Sheep: A Pilot Study. *Cartilage* 3:351-363. 10.1177/1947603512444597

76 Getgood AM, Kew SJ, Brooks R, Aberman H, Simon T, Lynn AK, and Rushton N. 2012b. Evaluation of early-stage osteochondral defect repair using a biphasic scaffold based on a collagen-glycosaminoglycan biopolymer in a caprine model. *Knee* 19:422-430. 10.1016/j.knee.2011.03.011

77 Ghosh P, Rameshbabu AP, and Dhara S. 2014. Citrate cross-linked gels with strain reversibility and viscoelastic behavior accelerate healing of osteochondral defects in a rabbit model. *Langmuir* 30:8442-8451. 10.1021/la500698v

78 Giordano M, Aulisa AG, Mastantuoni G, Gigante A, and Guzzanti V. 2011. Pridie's marrow stimulation technique combined with collagen matrix for cartilage repair. a study in a still growing sheep model. *International Journal of Immunopathology and Pharmacology* 24:101-106.

79 Gong L, Zhou X, Wu Y, Zhang Y, Wang C, Zhou H, Guo F, and Cui L. 2014. Proteomic analysis profile of engineered articular cartilage with chondrogenic differentiated adipose tissue-derived stem cells loaded polyglycolic acid mesh for weight-bearing area defect repair. *Tissue Engineering Part A* 20:575-587. 10.1089/ten.TEA.2013.0205

80 Gotterbarm T, Richter W, Jung M, Berardi Vilei S, Mainil-Varlet P, Yamashita T, and Breusch SJ. 2006. An in vivo study of a growth-factor enhanced, cell free, two-layered collagen-tricalcium phosphate in deep osteochondral defects. *Biomaterials* 27:3387-3395. 10.1016/j.biomaterials.2006.01.041

81 Guenther D, Liu C, Horstmann H, Krettek C, Jagodzinski M, and Haasper C. 2014. Near-infrared spectroscopy correlates with established histological scores in a miniature pig model of cartilage regeneration. *The Open Orthopaedics Journal* 8:93-99. 10.2174/1874325001408010093

82 Guo CA, Liu XG, Huo JZ, Jiang C, Wen XJ, and Chen ZR. 2007. Novel gene-modified-tissue engineering of cartilage using stable transforming growth factor-beta1-transfected mesenchymal stem cells grown on chitosan scaffolds. *Journal of Bioscience and Bioengineering* 103:547-556. 10.1263/jbb.103.547

83 Guo T, Yang TF, Xiao J, Long H, Chen YX, Pei FX, and Li YB. 2008a. Biomechanics study of polyvinyl alcohol hydrogel/nano-hydroxyapatite + polyamide66 in repair of articular cartilage and subchondral bone defects. *Journal of Clinical Rehabilitative Tissue Engineering Research* 12:1051-1054.

84 Guo T, Yang TF, Xiao J, Long H, Chen YX, Pei FX, and Li YB. 2008b. Repair of articular cartilage and subchondral defects with a new type biological composite material polyvinyl alcohol/nano-hydroxyapatite and polyamide 66. *Journal of Clinical Rehabilitative Tissue Engineering Research* 12:2623-2627.

85 Guo X, Park H, Young S, Kretlow JD, van den Beucken JJ, Baggett LS, Tabata Y, Kasper FK, Mikos AG, and Jansen JA. 2010. Repair of osteochondral defects with biodegradable hydrogel composites encapsulating marrow mesenchymal stem cells in a rabbit model. *Acta Biomaterialia* 6:39-47. 10.1016/j.actbio.2009.07.041

86 Gupta A, Bhat S, Jagdale PR, Chaudhari BP, Lidgren L, Gupta KC, and Kumar A. 2014. Evaluation of three-dimensional chitosan-agarose-gelatin cryogel scaffold for the repair of subchondral cartilage defects: an in vivo study in a rabbit model. *Tissue Engineering Part A* 20:3101-3111. 10.1089/ten.TEA.2013.0702

87 Guzman-Morales J, Lafantaisie-Favreau CH, Chen G, and Hoemann CD. 2014. Subchondral chitosan/blood implant-guided bone plate resorption and woven bone repair is coupled to hyaline cartilage regeneration from microdrill holes in aged rabbit knees. *Osteoarthritis Cartilage* 22:323-333. 10.1016/j.joca.2013.12.011

88 Hamanishi M, Nakasa T, Kamei N, Kazusa H, Kamei G, and Ochi M. 2013. Treatment of cartilage defects by subchondral drilling combined with covering with atelocollagen membrane induces osteogenesis in a rat model. *Journal of Orthopaedic Science* 18:627-635. 10.1007/s00776-013-0379-0

89 Han F, Yang X, Zhao J, Zhao Y, and Yuan X. 2015. Photocrosslinked layered gelatin-chitosan hydrogel with graded compositions for osteochondral defect repair. *Journal of Materials Science: Materials in Medicine* 26. 10.1007/s10856-015-5489-0

90 Han NB, Zhao JN, Zhang Y, He J, Lu X, Wang JL, and Guo T. 2009. Rabbit knee joint cartilage defect repaired by transforming growth factor-beta1-induced heparinized collagen/Chitosan scaffold binding with adipose-derived stem cells. *Journal of Clinical Rehabilitative Tissue Engineering Research* 13:6611-6616.

91 Hannink G, de Mulder EL, van Tienen TG, and Buma P. 2012. Effect of load on the repair of osteochondral defects using a porous polymer scaffold. *Journal of Biomedical Materials Research Part B* 100:2082-2089. 10.1002/jbm.b.32773

92 Hao JL, Wang YL, Sun ZY, Xia YY, Shi ZL, Zhu LY, and Feng WW. 2006. Repairing articular cartilage full-thickness defects with homograft of mesenchymal stem cells seeded onto novel scaffold composites hydroxyapatite/calcium polyphosphate/poly-L-lactide. *Chinese Journal of Clinical Rehabilitation* 10:64-67.

93 Hasegawa M, Sudo A, Shikinami Y, and Uchida A. 1999. Biological performance of a three-dimensional fabric as artificial cartilage in the repair of large osteochondral defects in rabbit. *Biomaterials* 20:1969-1975. 10.1016/S0142-9612(99)00106-4

94 He DD, Zeng LY, Xiang C, Wang YZ, Wang SD, Duan WP, Lu J, Wang CF, and Wei XC. 2012. Repairing articular cartilage defects in the knee of rabbits using type I/III-collagen-membrane combined with autologous bone marrow mesenchymal stem cells. *Chinese Journal of Tissue Engineering Research* 16:7031-7036.

95 Heikkila JT, Aho AJ, Yli-Urpo A, Andersson OH, Aho HJ, and Happonen RP. 1993. Bioactive glass versus hydroxylapatite in reconstruction of osteochondral defects in the rabbit. *Acta Orthopaedica Scandinavica* 64:678-682. 10.3109/17453679308994597

96 Ho ST, Hutmacher DW, Ekaputra AK, Hitendra D, and Hui JH. 2010. The evaluation of a biphasic osteochondral implant coupled with an electrospun membrane in a large animal model. *Tissue Engineering Part A* 16:1123-1141. 10.1089/ten.TEA.2009.0471

97 Hoemann CD, Chen G, Marchand C, Tran-Khanh N, Thibault M, Chevrier A, Sun J, Shive MS, Fernandes MJ, Poubelle PE, Centola M, and El-Gabalawy H. 2010. Scaffold-guided subchondral bone repair: implication of neutrophils and alternatively activated arginase-1+ macrophages. *The American Journal of Sports Medicine* 38:1845-1856. 10.1177/0363546510369547

98 Hoemann CD, Hurtig M, Rossomacha E, Sun J, Chevrier A, Shive MS, and Buschmann MD. 2005. Chitosan-glycerol phosphate/blood implants improve hyaline cartilage repair in ovine microfracture defects. *The Journal of Bone and Joint Surgery. American Volume* 87:2671-2686. 10.1016/j.joca.2006.06.015

99 Hoemann CD, Sun J, McKee MD, Chevrier A, Rossomacha E, Rivard GE, Hurtig M, and Buschmann MD. 2007. Chitosan-glycerol phosphate/blood implants elicit hyaline cartilage repair integrated with porous subchondral bone in microdrilled rabbit defects. *Osteoarthritis Cartilage* 15:78-89. 10.1016/j.joca.2006.06.015
100 Hogervorst T, Meijer DW, and Klopper PJ. 1992. The effect of a TCP-collagen implant on the healing of articular cartilage defects in the rabbit knee joint. *Journal of Biomaterials Applications* 3:251-258. 10.1002/jab.770030403
101 Holland TA, Bodde EW, Baggett LS, Tabata Y, Mikos AG, and Jansen JA. 2005. Osteochondral repair in the rabbit model utilizing bilayered, degradable oligo(poly(ethylene glycol) fumarate) hydrogel scaffolds. *Journal of Biomedical Materials Research Part A* 75:156-167. 10.1002/jbm.a.30379
102 Holland TA, Bodde EW, Cuijpers VM, Baggett LS, Tabata Y, Mikos AG, and Jansen JA. 2007. Degradable hydrogel scaffolds for in vivo delivery of single and dual growth factors in cartilage repair. *Osteoarthritis Cartilage* 15:187-197. 10.1016/j.joca.2006.07.006
103 Holmes M, Volz RG, and Chvapil M. 1975. Collagen sponge as a matrix for articular cartilage regeneration. *Surgical Forum* 26:511-513.
104 Howard D, Wardale J, Guehring MH, and Henson F. 2015. Delivering rhFGF-18 via a bilayer collagen membrane to enhance microfracture treatment of chondral defects in a large animal model. *Journal of Orthopaedic Research*. 10.1002/jor.22882
105 Huang H, Zhang X, Hu X, Shao Z, Zhu J, Dai L, Man Z, Yuan L, Chen H, Zhou C, and Ao Y. 2014. A functional biphasic biomaterial homing mesenchymal stem cells for in vivo cartilage regeneration. *Biomaterials* 35:9608-9619. 10.1016/j.biomaterials.2014.08.020
106 Huang X, Yang D, Yan W, Shi Z, Feng J, Gao Y, Weng W, and Yan S. 2007. Osteochondral repair using the combination of fibroblast growth factor and amorphous calcium phosphate/poly(l-lactic acid) hybrid materials. *Biomaterials* 28:3091-3100. 10.1016/j.biomaterials.2007.03.017
107 Huh SW, Shetty AA, Kim SJ, Kim YJ, Choi NY, Jun YJ, and Park IJ. 2014. The effect of platelet rich plasma combined with microfracture for the treatment of chondral defect in a rabbit knee. *Tissue Engineering and Regenerative Medicine* 11:178-185. 10.1007/s13770-013-1115-8
108 Hui JH, Ren X, Afizah MH, Chian KS, and Mikos AG. 2013. Oligo[poly(ethylene glycol)fumarate] hydrogel enhances osteochondral repair in porcine femoral condyle defects knee. *Clinical Orthopaedics and Related Research* 471:1174-1185. 10.1007/s11999-012-2487-0
109 Igarashi T, Iwasaki N, Kasahara Y, and Minami A. 2010. A cellular implantation system using an injectable ultra-purified alginate gel for repair of osteochondral defects in a rabbit model. *Journal of Biomedical Materials Research Part A* 94:844-855. 10.1002/jbm.a.32762
110 Igarashi T, Iwasaki N, Kawamura D, Kasahara Y, Tsukuda Y, Ohzawa N, Ito M, Izumisawa Y, and Minami A. 2012. Repair of articular cartilage defects with a novel injectable in situ forming material in a canine model. *Journal of Biomedical Materials Research Part A* 100:180-187. 10.1002/jbm.a.33248
111 Ikemura S, Hasegawa M, Iino T, Miyamoto K, Imanaka-Yoshida K, Yoshida T, and Sudo A. 2015. Effect of tenascin-C on the repair of full-thickness osteochondral defects of articular cartilage in rabbits. *Journal of Orthopaedic Research* 33:563-571. 10.1002/jor.22794
112 Im GI, Ahn JH, Kim SY, Choi BS, and Lee SW. 2010. A hyaluronate-atelocollagen/beta-tricalcium phosphate-hydroxyapatite biphasic scaffold for the repair of osteochondral defects: a porcine study. *Tissue Engineering Part A* 16:1189-1200. 10.1089/ten.TEA.2009.0540
113 Im GI, and Lee JH. 2010. Repair of osteochondral defects with adipose stem cells and a dual growth factor-releasing scaffold in rabbits. *Journal of Biomedical Materials Research Part B* 92:552-560. 10.1002/jbm.b.31552

114 Intzoglou KS, Mastrokalos DS, Korres DS, Papaparaskeva K, Koulalis D, and Babis GC. 2014. Donor's site evaluation after restoration with autografts or synthetic plugs in rabbits. *World Journal of Orthopaedics* 5:550-556. 10.5312/wjo.v5.i4.550
115 Ishii I, Mizuta H, Sei A, Hirose J, Kudo S, and Hiraki Y. 2007. Healing of full-thickness defects of the articular cartilage in rabbits using fibroblast growth factor-2 and a fibrin sealant. *The Journal of Bone and Joint Surgery* 89:693-700. 10.1302/0301-620X.89B5.18450
116 Ito Y, Adachi N, Nakamae A, Yanada S, and Ochi M. 2008. Transplantation of tissue-engineered osteochondral plug using cultured chondrocytes and interconnected porous calcium hydroxyapatite ceramic cylindrical plugs to treat osteochondral defects in a rabbit model. *Artificial Organs* 32:36-44. 10.1111/j.1525-1594.2007.00456.x
117 Ito Y, Ochi M, Adachi N, Sugawara K, Yanada S, Ikada Y, and Ronakorn P. 2005. Repair of osteochondral defect with tissue-engineered chondral plug in a rabbit model. *Arthroscopy* 21:1155-1163. 10.1016/j.arthro.2005.06.016
118 Jagodzinski M, Liu C, Guenther D, Burssens A, Petri M, Abedian R, Willbold E, Krettek C, Haasper C, and Witte F. 2014. Bone marrow-derived cell concentrates have limited effects on osteochondral reconstructions in the mini pig. *Tissue Engineering Part C* 20:215-226. 10.1089/ten.TEC.2013.0102
119 Jang KM, Lee JH, Park CM, Song HR, and Wang JH. 2014. Xenotransplantation of human mesenchymal stem cells for repair of osteochondral defects in rabbits using osteochondral biphasic composite constructs. *Knee Surgery, Sports Traumatology, Arthroscopy* 22:1434-1444. 10.1007/s00167-013-2426-y
120 Jansen EJ, Pieper J, Gijbels MJ, Guldemond NA, Riesle J, Van Rhijn LW, Bulstra SK, and Kuijer R. 2009. PEOT/PBT based scaffolds with low mechanical properties improve cartilage repair tissue formation in osteochondral defects. *Journal of Biomedical Materials Research Part A* 89:444-452. 10.1002/jbm.a.31986
121 Jansson V, Muller PE, Thal S, Arnholz C, Milz S, Koch KU, and Refior HJ. 2000. A new resorbable bone-cartilage replacement transplant. Results of an animal experiment study. *Orthopade* 29:151-157.
122 Jiang CC, Chiang H, Liao CJ, Lin YJ, Kuo TF, Shieh CS, Huang YY, and Tuan RS. 2007. Repair of porcine articular cartilage defect with a biphasic osteochondral composite. *Journal of Orthopaedic Research* 25:1277-1290. 10.1002/jor.20442
123 Jiang DM, Peng C, Guo ZP, and An H. 2010. Repairing articular cartilage and subchondral bone defects with polyurethane/nano-hydroxyapatite+polyamide66 composite. *Journal of Clinical Rehabilitative Tissue Engineering Research* 14:2883-2887. 10.3969/j.issn.1673-8225.2010.16.009
124 Jiang Y, Chen L, Zhang S, Tong T, Zhang W, Liu W, Xu G, Tuan RS, Heng BC, Crawford R, Xiao Y, and Ouyang HW. 2013. Incorporation of bioactive polyvinylpyrrolidone-iodine within bilayered collagen scaffolds enhances the differentiation and subchondral osteogenesis of mesenchymal stem cells. *Acta Biomaterialia* 9:8089-8098. 10.1016/j.actbio.2013.05.014
125 Jing B, Wang Z, Li B, Cheng Z, Kang Y, and Li Q. 2008. A preliminary study of high viscous chitosan/glycerol phosphate with demineralized bone matrix to repair cartilage defects in rabbits. *Chinese Journal of Reparative and Reconstructive Surgery* 22:1491-1494.
126 Jung M, Tuischer JS, Sergi C, Gotterbarm T, Pohl J, Richter W, and Simank HG. 2006. Local application of a collagen type I/hyaluronate matrix and growth and differentiation factor 5 influences the closure of osteochondral defects in a minipig model by enchondral ossification. *Growth Factors* 24:225-232. 10.1080/08977190600926969
127 Jung MR, Shim IK, Chung HJ, Lee HR, Park YJ, Lee MC, Yang YI, Do SH, and Lee SJ. 2012. Local BMP-7 release from a PLGA scaffolding-matrix for the repair of osteochondral defects in rabbits. *Journal of Controlled Release* 162:485-491. 10.1016/j.jconrel.2012.07.040
128 Jurgens WJ, Kroeze RJ, Zandieh-Doulabi B, van Dijk A, Renders GA, Smit TH, van Milligen FJ, Ritt MJ, and Helder MN. 2013. One-step surgical procedure for the treatment of

osteochondral defects with adipose-derived stem cells in a caprine knee defect: a pilot study. *BioResearch Open Access* 2:315-325. 10.1089/biores.2013.0024

129 Kandel RA, Grynpas M, Pilliar R, Lee J, Wang J, Waldman S, Zalzal P, and Hurtig M. 2006. Repair of osteochondral defects with biphasic cartilage-calcium polyphosphate constructs in a Sheep model. *Biomaterials* 27:4120-4131. 10.1016/j.biomaterials.2006.03.005

130 Kang H, Peng J, Lu S, Liu S, Zhang L, Huang J, Sui X, Zhao B, Wang A, Xu W, Luo Z, and Guo Q. 2012. In vivo cartilage repair using adipose-derived stem cell-loaded decellularized cartilage ECM scaffolds. *Journal of Tissue Engineering and Regenerative Medicine*. 10.1002/term.1538

131 Karagianes MT, Wheeler KR, and Nilles JL. 1975. Cartilage repair over porous metal implants. *Archives of Pathology and Laboratory Medicine* 99:398-400.

132 Kawaguchi Y, Kondo E, Kitamura N, Arakaki K, Tanaka Y, Munekata M, Nagai N, and Yasuda K. 2011. In vivo effects of isolated implantation of salmon-derived crosslinked atelocollagen sponge into an osteochondral defect. *Journal of Materials Science: Materials in Medicine* 22:397-404. 10.1007/s10856-010-4215-1

133 Kayakabe M, Tsutsumi S, Watanabe H, Kato Y, and Takagishi K. 2006. Transplantation of autologous rabbit BM-derived mesenchymal stromal cells embedded in hyaluronic acid gel sponge into osteochondral defects of the knee. *Cytotherapy* 8:343-353. 10.1080/14653240600845070

134 Kazemi D, Fakhrjou A, Mirzazadeh Dizaji V, and Khanzadeh Alishahi M. 2014. Effect of autologous platelet rich fibrin on the healing of experimental articular cartilage defects of the knee in an animal model. *BioMed Research International* 2014. 10.1155/2014/486436

135 Kim K, Lam J, Lu S, Spicer PP, Lueckgen A, Tabata Y, Wong ME, Jansen JA, Mikos AG, and Kasper FK. 2013. Osteochondral tissue regeneration using a bilayered composite hydrogel with modulating dual growth factor release kinetics in a rabbit model. *Journal of Controlled Release* 168:166-178. 10.1016/j.jconrel.2013.03.013

136 Kirker-Head CA, Van Sickle DC, Ek SW, and McCool JC. 2006. Safety of, and biological and functional response to, a novel metallic implant for the management of focal full-thickness cartilage defects: Preliminary assessment in an animal model out to 1 year. *Journal of Orthopaedic Research* 24:1095-1108. 10.1002/jor.20120

137 Kitamura N, Yasuda K, Ogawa M, Arakaki K, Kai S, Onodera S, Kurokawa T, and Gong JP. 2011. Induction of spontaneous hyaline cartilage regeneration using a double-network gel: efficacy of a novel therapeutic strategy for an articular cartilage defect. *The American Journal of Sports Medicine* 39:1160-1169. 10.1177/0363546511399383

138 Kon E, Delcogliano M, Filardo G, Fini M, Giavaresi G, Francioli S, Martin I, Pressato D, Arcangeli E, Quarto R, Sandri M, and Marcacci M. 2010a. Orderly osteochondral regeneration in a sheep model using a novel nano-composite multilayered biomaterial. *Journal of Orthopaedic Research* 28:116-124. 10.1002/jor.20958

139 Kon E, Filardo G, Delcogliano M, Fini M, Salamanna F, Giavaresi G, Martin I, and Marcacci M. 2010b. Platelet autologous growth factors decrease the osteochondral regeneration capability of a collagen-hydroxyapatite scaffold in a sheep model. *BMC Musculoskeletal Disorders* 11. 10.1186/1471-2474-11-220

140 Kon E, Filardo G, Robinson D, Eisman JA, Levy A, Zaslav K, Shani J, and Altschuler N. 2013. Osteochondral regeneration using a novel aragonite-hyaluronate bi-phasic scaffold in a goat model. *Knee Surgery, Sports Traumatology, Arthroscopy*. 10.1007/s00167-013-2467-2

141 Kon E, Mutini A, Arcangeli E, Delcogliano M, Filardo G, Nicoli Aldini N, Pressato D, Quarto R, Zaffagnini S, and Marcacci M. 2010c. Novel nanostructured scaffold for osteochondral regeneration: Pilot study in horses. *Journal of Tissue Engineering and Regenerative Medicine* 4:300-308. 10.1002/term.243

142 Kon M, and De Visser AC. 1981. A poly(HEMA) sponge for restoration of articular cartilage defects. *Plastic and Reconstructive Surgery* 67:289-293.

143 Kose GT, Korkusuz F, Ozkul A, Soysal Y, Ozdemir T, Yildiz C, and Hasirci V. 2005. Tissue engineered cartilage on collagen and PHBV matrices. *Biomaterials* 26:5187-5197. 10.1016/j.biomaterials.2005.01.037

144 Krych AJ, Wanivenhaus F, Ng KW, Doty S, Warren RF, and Maher SA. 2013. Matrix generation within a macroporous non-degradable implant for osteochondral defects is not enhanced with partial enzymatic digestion of the surrounding tissue: Evaluation in an in vivo rabbit model. *Journal of Materials Science: Materials in Medicine* 24:2429-2437. 1007/s10856-013-4999-x

145 Kubo M, Imai S, Fujimiya M, Isoya E, Ando K, Mimura T, and Matsusue Y. 2007. Exogenous collagen-enhanced recruitment of mesenchymal stem cells during rabbit articular cartilage repair. *Acta Orthopaedica* 78:845-855. 10.1080/17453670710014653

146 Kuo AC, Rodrigo JJ, Reddi AH, Curtiss S, Grotkopp E, and Chiu M. 2006. Microfracture and bone morphogenetic protein 7 (BMP-7) synergistically stimulate articular cartilage repair. *Osteoarthritis Cartilage* 14:1126-1135. 10.1016/j.joca.2006.04.004

147 Lafantaisie-Favreau CH, Guzman-Morales J, Sun J, Chen G, Harris A, Smith TD, Carli A, Henderson J, Stanish WD, and Hoemann CD. 2013. Subchondral pre-solidified chitosan/blood implants elicit reproducible early osteochondral wound-repair responses including neutrophil and stromal cell chemotaxis, bone resorption and repair, enhanced repair tissue integration and delayed matrix deposition. *BMC Musculoskeletal Disorders* 14. 10.1186/1471-2474-14-27

148 Lebourg M, Martinez-Diaz S, Garcia-Giralt N, Torres-Claramunt R, Ribelles JG, Vila-Canet G, and Monllau J. 2014. Cell-free cartilage engineering approach using hyaluronic acid-polycaprolactone scaffolds: A study in vivo. *Journal of Biomaterials Applications* 28:1304-1315. 10.1177/0885328213507298

149 Lee JC, Lee SY, Min HJ, Han SA, Jang J, Lee S, Seong SC, and Lee MC. 2012. Synovium-derived mesenchymal stem cells encapsulated in a novel injectable gel can repair osteochondral defects in a rabbit model. *Tissue Engineering Part A* 18:2173-2186. 10.1089/ten.TEA.2011.0643

150 Lee JC, Min HJ, Park HJ, Lee S, Seong SC, and Lee MC. 2013. Synovial membrane-derived mesenchymal stem cells supported by platelet-rich plasma can repair osteochondral defects in a rabbit model. *Arthroscopy* 29:1034-1046. 10.1016/j.arthro.2013.02.026

151 Lee JM, Kim JD, Oh EJ, Oh SH, Lee JH, and Im GI. 2014. PD98059-impregnated functional plga scaffold for direct tissue engineering promotes chondrogenesis and prevents hypertrophy from mesenchymal stem cells. *Tissue Engineering Part A* 20:982-991. 10.1089/ten.TEA.2013.0290

152 Li Q, Tang JC, Sun ZY, Wang SK, and Li WZ. 2008. Repairing full-thickness articular cartilage defects with homograft of mesenchymal stem cells seeded onto cancellous demineralized bone matrix. *Journal of Clinical Rehabilitative Tissue Engineering Research* 12:8943-8947.

153 Li G, Fu N, Xie J, Fu Y, Deng S, Cun X, Wei X, Peng Q, Cai X, and Lin Y. 2015a. Poly(3-hydroxybutyrate-co-4-hydroxybutyrate) based electrospun 3D scaffolds for delivery of autogeneic chondrocytes and adipose-derived stem cells: Evaluation of cartilage defects in rabbit. *Journal of Biomedical Nanotechnology* 11:105-116. 10.1166/jbn.2015.2053

154 Li X, Li Y, Zuo Y, Qu D, Liu Y, Chen T, Jiang N, Li H, and Li J. 2015b. Osteogenesis and chondrogenesis of biomimetic integrated porous PVA/gel/V-n-HA/pa6 scaffolds and BMSCs construct in repair of articular osteochondral defect. *Journal of Biomedical Materials Research Part A*. 10.1002/jbm.a.35452

155 Lim CT, Ren X, Afizah MH, Tarigan-Panjaitan S, Yang Z, Wu Y, Chian KS, Mikos AG, and Hui JH. 2013. Repair of osteochondral defects with rehydrated freeze-dried oligo[poly(ethylene glycol) fumarate] hydrogels seeded with bone marrow mesenchymal stem cells in a porcine model. *Tissue Engineering Part A* 19:1852-1861. 10.1089/ten.TEA.2012.0621

156 Lin J, Wang R, and Cheng L. 2006. Articular cartilage defects repaired with homograft of mesenchymal stem cells seeded onto medical collagen membrane of guided tissue regeneration. *Chinese Journal of Reparative and Reconstructive Surgery* 20:1229-1234.

157 Liu J, Nie H, Xu Z, Niu X, Guo S, Yin J, Guo F, Li G, Wang Y, and Zhang C. 2014a. The effect of 3D nanofibrous scaffolds on the chondrogenesis of induced pluripotent stem cells and their application in restoration of cartilage defects. *PLoS One* 9:e111566. 10.1371/journal.pone.0111566

158 Liu M, Xiang Z, Pei F, Huang F, Cen S, Zhong G, Fan H, Xiao Y, Sun J, and Gao Y. 2010. Repairing defects of rabbit articular cartilage and subchondral bone with biphasic scaffold combined bone marrow stromal stem cells. *Chinese Journal of Reparative and Reconstructive Surgery* 24:87-93.

159 Liu PF, Guo L, Zhao DW, Zhang ZJ, Kang K, Zhu RP, and Yuan XL. 2014b. Study of human acellular amniotic membrane loading bone marrow mesenchymal stem cells in repair of articular cartilage defect in rabbits. *Genetics and Molecular Research* 13:7992-8001. 10.4238/2014.September.29.12

160 Liu Y, Shu XZ, and Prestwich GD. 2006. Osteochondral defect repair with autologous bone marrow-derived mesenchymal stem cells in an injectable, in situ, cross-linked synthetic extracellular matrix. *Tissue Engineering* 12:3405-3416. 10.1089/ten.2006.12.3405

161 Lohan A, Marzahn U, El Sayed K, Haisch A, Muller RD, Kohl B, Stolzel K, Ertel W, John T, and Schulze-Tanzil G. 2014. Osteochondral articular defect repair using auricle-derived autologous chondrocytes in a rabbit model. *Annals of Anatomy* 196:317-326. 10.1016/j.aanat.2014.03.002

162 Loken S, Jakobsen RB, Aroen A, Heir S, Shahdadfar A, Brinchmann JE, Engebretsen L, and Reinholt FP. 2008. Bone marrow mesenchymal stem cells in a hyaluronan scaffold for treatment of an osteochondral defect in a rabbit model. *Knee Surgery, Sports Traumatology, Arthroscopy* 16:896-903. 10.1007/s00167-008-0566-2

163 Lopiz-Morales Y, Abarrategi A, Ramos V, Moreno-Vicente C, Lopez-Duran L, Lopez-Lacomba JL, and Marco F. 2010. In vivo comparison of the effects of rhBMP-2 and rhBMP-4 in osteochondral tissue regeneration. *European Cells and Materials* 20:367-378.

164 Lu HD, Cai DZ, Liu Q, Wang YJ, and Zheng YD. 2005. Experimental study of rabbit knee joint articular cartilage defects repaired with transplantation of polyvinyl alcohol/hydroxyapatite composite hydrogel. *Journal of Clinical Rehabilitative Tissue Engineering Research* 9:184-186.

165 Lu S, Lam J, Trachtenberg JE, Lee EJ, Seyednejad H, van den Beucken JJ, Tabata Y, Wong ME, Jansen JA, Mikos AG, and Kasper FK. 2014. Dual growth factor delivery from bilayered, biodegradable hydrogel composites for spatially-guided osteochondral tissue repair. *Biomaterials* 35:8829-8839. 10.1016/j.biomaterials.2014.07.006

166 Lubiatowski P, Kruczynski J, Gradys A, Trzeciak T, and Jaroszewski J. 2006. Articular cartilage repair by means of biodegradable scaffolds. *Transplantation Proceedings* 38:320-322. 10.1016/j.transproceed.2005.12.012

167 Luo Z, Jiang L, Xu Y, Li H, Xu W, Wu S, Wang Y, Tang Z, Lv Y, and Yang L. 2015. Mechano growth factor (MGF) and transforming growth factor (TGF)-beta3 functionalized silk scaffolds enhance articular hyaline cartilage regeneration in rabbit model. *Biomaterials* 52:463-475. 10.1016/j.biomaterials.2015.01.001

168 Maehara H, Sotome S, Yoshii T, Torigoe I, Kawasaki Y, Sugata Y, Yuasa M, Hirano M, Mochizuki N, Kikuchi M, Shinomiya K, and Okawa A. 2010. Repair of large osteochondral defects in rabbits using porous hydroxyapatite/collagen (HAp/Col) and fibroblast growth factor-2 (FGF-2). *Journal of Orthopaedic Research* 28:677-686. 10.1002/jor.21032

169 Marchand C, Chen G, Tran-Khanh N, Sun J, Chen H, Buschmann MD, and Hoemann CD. 2012. Microdrilled cartilage defects treated with thrombin-solidified chitosan/blood implant regenerate a more hyaline, stable, and structurally integrated osteochondral unit compared to drilled controls. *Tissue Engineering Part A* 18:508-519. 10.1089/ten.TEA.2011.0178

170 Marmotti A, Bruzzone M, Bonasia DE, Castoldi F, Rossi R, Piras L, Maiello A, Realmuto C, and Peretti GM. 2012. One-step osteochondral repair with cartilage fragments in a composite scaffold. *Knee Surgery, Sports Traumatology, Arthroscopy* 20:2590-2601. 10.1007/s00167-012-1920-y

171 Marmotti A, Bruzzone M, Bonasia DE, Castoldi F, Von Degerfeld MM, Bignardi C, Mattia S, Maiello A, Rossi R, and Peretti GM. 2013. Autologous cartilage fragments in a composite scaffold for one stage osteochondral repair in a goat model. *European Cells and Materials* 26:15-32.

172 Martinez-Carranza N, Berg HE, Lagerstedt AS, Nurmi-Sandh H, Schupbach P, and Ryd L. 2014. Fixation of a double-coated titanium-hydroxyapatite focal knee resurfacing implant: a 12-month study in sheep. *Osteoarthritis Cartilage* 22:836-844. 10.1016/j.joca.2014.03.019

173 Martinez-Diaz S, Garcia-Giralt N, Lebourg M, Gomez-Tejedor JA, Vila G, Caceres E, Benito P, Pradas MM, Nogues X, Ribelles JL, and Monllau JC. 2010. In vivo evaluation of 3-dimensional polycaprolactone scaffolds for cartilage repair in rabbits. *The American Journal of Sports Medicine* 38:509-519. 10.1177/0363546509352448

174 Martins EA, Michelacci YM, Baccarin RY, Cogliati B, and Silva LC. 2014. Evaluation of chitosan-GP hydrogel biocompatibility in osteochondral defects: an experimental approach. *BMC Veterinary Research* 10:197. 10.1186/s12917-014-0197-4

175 Masuoka K, Asazuma T, Hattori H, Yoshihara Y, Sato M, Matsumura K, Matsui T, Takase B, Nemoto K, and Ishihara M. 2006. Tissue engineering of articular cartilage with autologous cultured adipose tissue-derived stromal cells using atelocollagen honeycomb-shaped scaffold with a membrane sealing in rabbits. *Journal of Biomedical Materials Research Part B* 79:25-34. 10.1002/jbm.b.30507

176 Matsuda H, Kitamura N, Kurokawa T, Arakaki K, Gong JP, Kanaya F, and Yasuda K. 2013. Influence of the gel thickness on in vivo hyaline cartilage regeneration induced by double-network gel implanted at the bottom of a large osteochondral defect: short-term results. *BMC Musculoskeletal Disorders* 14. 10.1186/1471-2474-14-50

177 Mazaki T, Shiozaki Y, Yamane K, Yoshida A, Nakamura M, Yoshida Y, Zhou D, Kitajima T, Tanaka M, Ito Y, Ozaki T, and Matsukawa A. 2014. A novel, visible light-induced, rapidly cross-linkable gelatin scaffold for osteochondral tissue engineering. *Scientific Reports* 4:4457. 10.1038/srep04457

178 Messner K. 1993. Hydroxylapatite supported Dacron plugs for repair of isolated full-thickness osteochondral defects of the rabbit femoral condyle: mechanical and histological evaluations from 6-48 weeks. *Journal of Biomedical Materials Research* 27:1527-1532. 10.1002/jbm.820271209

179 Messner K. 1994. Durability of artificial implants for repair of osteochondral defects of the medial femoral condyle in rabbits. *Biomaterials* 15:657-664. 10.1016/0142-9612(94)90163-5

180 Miao ZN, Pan YH, Zhu JZ, Qian HG, and Zhao JD. 2008. Constructing tissue engineered cartilage with silk fibroin compound bone marrow mesenchymal stem cells. *Journal of Clinical Rehabilitative Tissue Engineering Research* 12:5243-5247.

181 Mierisch CM, Cohen SB, Jordan LC, Robertson PG, Balian G, and Diduch DR. 2002. Transforming growth factor-beta in calcium alginate beads for the treatment of articular cartilage defects in the rabbit. *Arthroscopy* 18:892-900. 10.1053/jars.2002.36117

182 Miljkovic ND, Lin YC, Cherubino M, Minteer D, and Marra KG. 2009. A novel injectable hydrogel in combination with a surgical sealant in a rat knee osteochondral defect model. *Knee Surgery, Sports Traumatology, Arthroscopy* 17:1326-1331. 10.1007/s00167-009-0881-2

183 Miller RE, Grodzinsky AJ, Barrett MF, Hung HH, Frank EH, Werpy NM, McIlwraith CW, and Frisbie DD. 2014. Effects of the combination of microfracture and self-assembling peptide filling on the repair of a clinically relevant trochlear defect in an equine model. *The Journal of Bone and Joint Surgery. American Volume* 96:1601-1609. 10.2106/JBJS.M.01408

184 Miller RE, Grodzinsky AJ, Vanderploeg EJ, Lee C, Ferris DJ, Barrett MF, Kisiday JD, and Frisbie DD. 2010. Effect of self-assembling peptide, chondrogenic factors, and bone marrow-derived stromal cells on osteochondral repair. *Osteoarthritis Cartilage* 18:1608-1619. 10.1016/j.joca.2010.09.004

185 Mimura T, Imai S, Kubo M, Isoya E, Ando K, Okumura N, and Matsusue Y. 2008. A novel exogenous concentration-gradient collagen scaffold augments full-thickness articular cartilage repair. *Osteoarthritis Cartilage* 16:1083-1091. 10.1016/j.joca.2008.02.003

186 Mimura T, Imai S, Okumura N, Li L, Nishizawa K, Araki S, Ueba H, Kubo M, Mori K, and Matsusue Y. 2011. Spatiotemporal control of proliferation and differentiation of bone marrow-derived mesenchymal stem cells recruited using collagen hydrogel for repair of articular cartilage defects. *Journal of Biomedical Materials Research Part B* 98 B:360-368. 10.1002/jbm.b.31859

187 Mohan N, Dormer NH, Caldwell KL, Key VH, Berkland CJ, and Detamore MS. 2011. Continuous gradients of material composition and growth factors for effective regeneration of the osteochondral interface. *Tissue Engineering Part A* 17:2845-2855. 10.1089/ten.tea.2011.0135

188 Mori H, Kondo E, Kawaguchi Y, Kitamura N, Nagai N, Iida H, and Yasuda K. 2013. Development of a salmon-derived crosslinked atelocollagen sponge disc containing osteogenic protein-1 for articular cartilage regeneration: In vivo evaluations with rabbits. *BMC Musculoskeletal Disorders* 14. 10.1186/1471-2474-14-174

189 Mrosek EH, Schagemann JC, Chung HW, Fitzsimmons JS, Yaszemski MJ, Mardones RM, O'Driscoll SW, and Reinholz GG. 2010. Porous tantalum and poly-epsilon-caprolactone biocomposites for osteochondral defect repair: preliminary studies in rabbits. *Journal of Orthopaedic Research* 28:141-148. 10.1002/jor.20983

190 Muller PE, Schrimpf F, Milz S, Kircher J, Durr HR, Wegener B, Pellengahr C, and Jansson V. 2006. Repair of osteochondral defects in the knee by resorbable bioimplants in a rabbit model. *Acta Orthopaedica* 77:981-985. 10.1080/17453670610013321

191 Munirah S, Samsudin OC, Chen HC, Salmah SH, Aminuddin BS, and Ruszymah BH. 2007. Articular cartilage restoration in load-bearing osteochondral defects by implantation of autologous chondrocyte-fibrin constructs: an experimental study in sheep. *The Journal of Bone and Joint Surgery. British Volume* 89:1099-1109. 10.1302/0301-620x.89b8.18451

192 Nagura I, Fujioka H, Kokubu T, Makino T, Sumi Y, and Kurosaka M. 2007. Repair of osteochondral defects with a new porous synthetic polymer scaffold. *The Journal of Bone and Joint Surgery. British Volume* 89:258-264. 10.1302/0301-620X.89B2.17754

193 Necas A, Planka L, Srnec R, Crha M, Hlucilova J, Klima J, Stary D, Kren L, Amler E, Vojtova L, Jancar J, and Gal P. 2010. Quality of newly formed cartilaginous tissue in defects of articular surface after transplantation of mesenchymal stem cells in a composite scaffold based on collagen I with chitosan micro- and nanofibres. *Physiological Research* 59:605-614.

194 Needham CJ, Shah SR, Dahlin RL, Kinard LA, Lam J, Watson BM, Lu S, Kasper FK, and Mikos AG. 2014. Osteochondral tissue regeneration through polymeric delivery of DNA encoding for the SOX trio and RUNX2. *Acta Biomaterialia* 10:4103-4112. 10.1016/j.actbio.2014.05.011

195 Nettles DL, Kitaoka K, Hanson NA, Flahiff CM, Mata BA, Hsu EW, Chilkoti A, and Setton LA. 2008. In situ crosslinking elastin-like polypeptide gels for application to articular cartilage repair in a goat osteochondral defect model. *Tissue Engineering Part A* 14:1133-1140. 10.1089/tea.2007.0245

196 Niederauer GG, M AS, Leatherbury NC, Korvick DL, Harroff Jr HH, Ehler WC, Dunn CJ, and Kieswetter K. 2000. Evaluation of multiphase implants for repair of focal osteochondral defects in goats. *Biomaterials* 21:2561-2574. 10.1016/S0142-9612(00)00124-1

197 Nixon AJ, Fortier LA, Williams J, and Mohammed H. 1999. Enhanced repair of extensive articular defects by insulin-like growth factor-I-laden fibrin composites. *Journal of Orthopaedic Research* 17:475-487. 10.1002/jor.1100170404

198 Noguchi T, Oka M, Fujino M, Neo M, and Yamamuro T. 1994. Repair of osteochondral defects with grafts of cultured chondrocytes. Comparison of allografts and isografts. *Clinical Orthopaedics and Related Research* 302:251-258.

199 Ogawa M, Kitamura N, Kurokawa T, Arakaki K, Tanaka Y, Gong JP, and Yasuda K. 2012. Poly(2-acrylamido-2-methylpropanesulfonic acid) gel induces articular cartilage regeneration in

vivo: Comparisons of the induction ability between single- and double-network gels. *Journal of Biomedical Materials Research Part A* 100 A:2244-2251. 10.1002/jbm.a.34165
200 Oliveira JT, Gardel LS, Rada T, Martins L, Gomes ME, and Reis RL. 2010. Injectable gellan gum hydrogels with autologous cells for the treatment of rabbit articular cartilage defects. *Journal of Orthopaedic Research* 28:1193-1199. 10.1002/jor.21114
201 Oshima Y, Harwood FL, Coutts RD, Kubo T, and Amiel D. 2009. Variation of mesenchymal cells in polylactic acid scaffold in an osteochondral repair model. *Tissue Engineering Part C* 15:595-604. 10.1089/ten.TEC.2008.0487
202 Oshima Y, Watanabe N, Matsuda K, Takai S, Kawata M, and Kubo T. 2005. Behavior of transplanted bone marrow-derived GFP mesenchymal cells in osteochondral defect as a simulation of autologous transplantation. *Journal of Histochemistry & Cytochemistry* 53:207-216. 10.1369/jhc.4A6280.2005
203 Paletta GA, Arnoczky SP, and Warren RF. 1992. The repair of osteochondral defects using an exogenous fibrin clot. An experimental study in dogs. *The American Journal of Sports Medicine* 20:725-731. 10.1177/036354659202000614
204 Pei M, He F, Boyce BM, and Kish VL. 2009. Repair of full-thickness femoral condyle cartilage defects using allogeneic synovial cell-engineered tissue constructs. *Osteoarthritis Cartilage* 17:714-722. 10.1016/j.joca.2008.11.017
205 Perka C, Schultz O, Sittinger M, and Zippel H. 2000. Chondrocyte transplantation in PGLA/polydioxanone fleece. *Orthopade* 29:112-119.
206 Perka C, Schultz O, Spitzer RS, and Lindenhayn K. 2000. The influence of transforming growth factor beta1 on mesenchymal cell repair of full-thickness cartilage defects. *Journal of Biomedical Materials Research* 52(3): 543-552.
207 Pilliar RM, Kandel RA, Grynpas MD, Zalzal P, and Hurtig M. 2007. Osteochondral defect repair using a novel tissue engineering approach: Sheep model study. *Technology and Health Care* 15:47-56.
208 Ponticiello MS, Schinagl RM, Kadiyala S, and Barry FP. 2000. Gelatin-based resorbable sponge as a carrier matrix for human mesenchymal stem cells in cartilage regeneration therapy. *Journal of Biomedical Materials Research* 52:246-255. 10.1002/1097-4636(200011)52:23.0.CO;2-W
209 Qi H, Sun L, Chen L, Tao JF, and Jiang J. 2010. Demineralized bone matrix compounded with biological fibrin glue for repairing rabbit cartilage defects. *Journal of Clinical Rehabilitative Tissue Engineering Research* 14:4589-4593. 10.3969/j.issn.1673-8225.2010.25.008
210 Qi Y, Du Y, Li W, Dai X, Zhao T, and Yan W. 2012. Cartilage repair using mesenchymal stem cell (MSC) sheet and MSCs-loaded bilayer PLGA scaffold in a rabbit model. *Knee Surgery, Sports Traumatology, Arthroscopy*. 10.1007/s00167-012-2256-3
211 Qiu YS, Shahgaldi BF, Revell WJ, and Heatley FW. 2002. Evaluation of Gateshead carbon fibre rod as an implant material for repair of osteochondral defects: a morphological and mechanical study in the rabbit knee. *Biomaterials* 23:3943-3955.
212 Ravanetti F, Galli C, Manfredi E, Cantoni A, Scarpa E, Macaluso G, and Cacchioli A. 2015. Chitosan-based scaffold modified with D-(+) raffinose for cartilage repair: an in vivo study. *Journal of Negative Results in BioMedicine* 14:2. 10.1186/s12952-014-0021-5
213 Re'Em T, Witte F, Willbold E, Ruvinov E, and Cohen S. 2012. Simultaneous regeneration of articular cartilage and subchondral bone induced by spatially presented TGF-beta and BMP-4 in a bilayer affinity binding system. *Acta Biomaterialia* 8:3283-3293. 10.1016/j.actbio.2012.05.014
214 Reyes R, Delgado A, Sanchez E, Fernandez A, Hernandez A, and Evora C. 2012. Repair of an osteochondral defect by sustained delivery of BMP-2 or TGFbeta1 from a bilayered alginate-PLGA scaffold. *Journal of Tissue Engineering and Regenerative Medicine*. 10.1002/term.1549
215 Reyes R, Delgado A, Solis R, Sanchez E, Hernandez A, San Roman J, and Evora C. 2014. Cartilage repair by local delivery of transforming growth factor-beta1 or bone morphogenetic

protein-2 from a novel, segmented polyurethane/polylactic-co-glycolic bilayered scaffold. *Journal of Biomedical Materials Research Part A* 102:1110-1120. 10.1002/jbma.34769
216 Reyes R, Pec MK, Sanchez E, del Rosario C, Delgado A, and Evora C. 2013. Comparative, osteochondral defect repair: stem cells versus chondrocytes versus bone morphogenetic protein-2, solely or in combination. *European Cells and Materials* 25:351-365; discussion 365.
217 Rudert M, Wilms U, Hoberg M, and Wirth CJ. 2005. Cell-based treatment of osteochondral defects in the rabbit knee with natural and synthetic matrices: cellular seeding determines the outcome. *Archives of Orthopaedic and Trauma Surgery* 125:598-608. 10.1007/s00402-005-0008-2
218 Saha S, Kundu B, Kirkham J, Wood D, Kundu SC, and Yang XB. 2013. Osteochondral tissue engineering in vivo: A comparative study using layered silk fibroin scaffolds from mulberry and nonmulberry silkworms. *PLoS One* 8. 10.1371/journal.pone.0080004
219 Sakata R, Kokubu T, Mifune Y, Inui A, Nishimoto H, Fujioka H, Kuroda R, and Kurosaka M. 2014. A new bioabsorbable cotton-textured synthetic polymer scaffold for osteochondral repair. *IInternational Orthopaedics*. 10.1007/s00264-013-2253-2
220 Sakata R, Kokubu T, Nagura I, Toyokawa N, Inui A, Fujioka H, and Kurosaka M. 2012. Localization of vascular endothelial growth factor during the early stages of osteochondral regeneration using a bioabsorbable synthetic polymer scaffold. *Journal of Orthopaedic Research* 30:252-259. 10.1002/jor.21502
221 Schaefer D, Martin I, Jundt G, Seidel J, Heberer M, Grodzinsky A, Bergin I, Vunjak-Novakovic G, and Freed LE. 2002. Tissue-engineered composites for the repair of large osteochondral defects. *Arthritis & Rheumatology* 46:2524-2534. 10.1002/art.10493
222 Schagemann JC, Erggelet C, Chung HW, Lahm A, Kurz H, and Mrosek EH. 2009. Cell-laden and cell-free biopolymer hydrogel for the treatment of osteochondral defects in a sheep model. *Tissue Engineering Part A* 15:75-82. 10.1089/ten.tea.2008.0087
223 Schleicher I, Lips KS, Sommer U, Schappat I, Martin AP, Szalay G, Hartmann S, and Schnettler R. 2013. Biphasic scaffolds for repair of deep osteochondral defects in a sheep model. *Journal of Surgical Research* 183:184-192. 10.1016/j.jss.2012.11.036
224 Schlichting K, Schell H, Kleemann RU, Schill A, Weiler A, Duda GN, and Epari DR. 2008. Influence of scaffold stiffness on subchondral bone and subsequent cartilage regeneration in an ovine model of osteochondral defect healing. *The American Journal of Sports Medicine* 36:2379-2391. 10.1177/0363546508322899
225 Seo JP, Tanabe T, Tsuzuki N, Haneda S, Yamada K, Furuoka H, Tabata Y, and Sasaki N. 2013. Effects of bilayer gelatin/beta-tricalcium phosphate sponges loaded with mesenchymal stem cells, chondrocytes, bone morphogenetic protein-2, and platelet rich plasma on osteochondral defects of the talus in horses. *Research in Veterinary Science* 95:1210-1216. 10.1016/j.rvsc.2013.08.016
226 Seol D, Magnetta MJ, Ramakrishnan PS, Kurriger GL, Choe H, Jang K, Martin JA, and Lim TH. 2013. Biocompatibility and preclinical feasibility tests of a temperature-sensitive hydrogel for the purpose of surgical wound pain control and cartilage repair. *Journal of Biomedical Materials Research Part B* 101:1508-1515. 10.1002/jbmb.32981
227 Shahgaldi BF. 1998. Coral graft restoration of osteochondral defects. *Biomaterials* 19:205-213. 10.1016/S0142-9612(98)80002-1
228 Shao X, Goh JC, Hutmacher DW, Lee EH, and Zigang G. 2006a. Repair of large articular osteochondral defects using hybrid scaffolds and bone marrow-derived mesenchymal stem cells in a rabbit model. *Tissue Engineering* 12:1539-1551. 10.1089/ten.2006.12.1539
229 Shao XX, Hutmacher DW, Ho ST, Goh JC, and Lee EH. 2006b. Evaluation of a hybrid scaffold/cell construct in repair of high-load-bearing osteochondral defects in rabbits. *Biomaterials* 27:1071-1080. 10.1016/j.biomaterials.2005.07.040
230 Shao Z, Zhang X, Pi Y, Wang X, Jia Z, Zhu J, Dai L, Chen W, Yin L, Chen H, Zhou C, and Ao Y. 2012. Polycaprolactone electrospun mesh conjugated with an MSC affinity peptide for MSC homing in vivo. *Biomaterials* 33:3375-3387. 10.1016/j.biomaterials.2012.01.033

231 Shi J, Zhang X, Zeng X, Zhu J, Pi Y, Zhou C, and Ao Y. 2012. One-step articular cartilage repair: Combination of in situ bone marrow stem cells with cell-free poly(L-lactic-co-glycolic acid) scaffold in a rabbit model. *Orthopedics* 35:e665-e671. 10.3928/01477447-20120426-20

232 Shim IK, Yook YJ, Lee SY, Lee SH, Park KD, Lee MC, and Lee SJ. 2008. Healing of articular cartilage defects treated with a novel drug-releasing rod-type implant after microfracture surgery. *Journal of Controlled Release* 129:187-191. 10.1016/j.jconrel.2008.04.003

233 Shimomura K, Moriguchi Y, Ando W, Nansai R, Fujie H, Hart DA, Gobbi A, Kita K, Horibe S, Shino K, Yoshikawa H, and Nakamura N. 2014. Osteochondral Repair Using a Scaffold-Free Tissue-Engineered Construct Derived from Synovial Mesenchymal Stem Cells and a Hydroxyapatite-Based Artificial Bone. *Tissue Engineering Part A*. 10.1089/ten.tea.2013.0414

234 Solchaga LA, Gao J, Dennis JE, Awadallah A, Lundberg M, Caplan AI, and Goldberg VM. 2002. Treatment of osteochondral defects with autologous bone marrow in a hyaluronan-based delivery vehicle. *Tissue Engineering* 8:333-347. 10.1089/107632702753725085

235 Solchaga LA, Temenoff JS, Gao J, Mikos AG, Caplan AI, and Goldberg VM. 2005. Repair of osteochondral defects with hyaluronan- and polyester-based scaffolds. *Osteoarthritis Cartilage* 13:297-309. 10.1016/j.joca.2004.12.016

236 Solchaga LA, Yoo JU, Lundberg M, Dennis JE, Huibregtse BA, Goldberg VM, and Caplan AI. 2000. Hyaluronan-based polymers in the treatment of osteochondral defects. *Journal of Orthopaedic Research* 18:773-780. 10.1002/jor.1100180515

237 Song ZM, Zhang SK, Liu Y, Ren Q, and Fu CF. 2006. Transgeneic allogenic tissue-engineered cartilage in repairing the full thickness defects of knee in rabbits. *Chinese Journal of Clinical Rehabilitation* 10:60-63.

238 Sosio C, Di Giancamillo A, Deponti D, Gervaso F, Scalera F, Melato M, Campagnol M, Boschetti F, Nonis A, Domeneghini C, Sannino A, and Peretti GM. 2015. Osteochondral repair by a novel interconnecting collagen-hydroxyapatite substitute: a large-animal study. *Tissue Engineering Part A* 21:704-715. 10.1089/ten.TEA.2014.0129

239 Sotoudeh A, Jahanshahi A, Takhtfoolad MA, Bazazan A, Ganjali A, and Harati MP. 2013. Study on nano-structured hydroxyapatite/zirconia stabilized yttria on healing of articular cartilage defect in rabbit. *Acta Cirurgica Brasileira* 28:340-345. 10.1590/S0102-86502013000500004

240 Speer DP, Chvapil M, Volz RG, and Holmes MD. 1979. Enhancement of healing in osteochondral defects by collagen sponge implants. *Clinical Orthopaedics and Related Research* 144:326-335.

241 Streitparth F, Schottle P, Schlichting K, Schell H, Fischbach F, Denecke T, Duda GN, and Schroder RJ. 2009. Osteochondral defect repair after implantation of biodegradable scaffolds: indirect magnetic resonance arthrography and histopathologic correlation. *Acta Radiologica* 50:765-774. 10.1080/02841850902980272

242 Sukegawa A, Iwasaki N, Kasahara Y, Onodera T, Igarashi T, and Minami A. 2012. Repair of rabbit osteochondral defects by an acellular technique with an ultrapurified alginate gel containing stromal cell-derived factor-1. *Tissue Engineering Part A* 18:934-945. 10.1089/ten.TEA.2011.0380

243 Sun Y, Feng Y, Zhang CQ, Chen SB, and Cheng XG. 2010. The regenerative effect of platelet-rich plasma on healing in large osteochondral defects. *International Orthopaedics* 34:589-597. 10.1007/s00264-009-0793-2

244 Suominen E, Aho AJ, Vedel E, Kangasniemi I, Uusipaikka E, and Yli-Urpo A. 1996. Subchondral bone and cartilage repair with bioactive glasses, hydroxyapatite, and hydroxyapatite-glass composite. *Journal of Biomedical Materials Research* 32:543-551. 10.1002/(SICI)1097-4636(199612)32:4<543::AID-JBM7>3.0.CO;2-R

245 Tamai N, Myoui A, Hirao M, Kaito T, Ochi T, Tanaka J, Takaoka K, and Yoshikawa H. 2005. A new biotechnology for articular cartilage repair: Subchondral implantation of a composite of interconnected porous hydroxyapatite, synthetic polymer (PLA-PEG), and bone morphogenetic protein-2 (rhBMP-2). *Osteoarthritis Cartilage* 13:405-417. 10.1016/j.joca.2004.12.014

246 Tan WC, Zha ZG, Zhang JQ, Zheng LH, Liang YZ, Xia JS, Huang XP, Wu H, and Lin HS. 2011. Animal-origin osteochondral scaffold combined with bone marrow mesenchymal stem cells/chondrocytes for repair of composite osteochondral defects in rabbit knee joints. *Journal of Clinical Rehabilitative Tissue Engineering Research* 15:2265-2269.
247 Tanaka T, Komaki H, Chazono M, and Fujii K. 2005. Use of a biphasic graft constructed with chondrocytes overlying a beta-tricalcium phosphate block in the treatment of rabbit osteochondral defects. *Tissue Engineering* 11:331-339. 10.1089/ten.2005.11.331
248 Tatebe M, Nakamura R, Kagami H, Okada K, and Ueda M. 2005. Differentiation of transplanted mesenchymal stem cells in a large osteochondral defect in rabbit. *Cytotherapy* 7:520-530. 10.1080/14653240500361350
249 Toyokawa N, Fujioka H, Kokubu T, Nagura I, Inui A, Sakata R, Satake M, Kaneko H, and Kurosaka M. 2010. Electrospun Synthetic Polymer Scaffold for Cartilage Repair Without Cultured Cells in an Animal Model. *Arthroscopy* 26:375-383. 10.1016/j.arthro.2009.08.006
250 von Schroeder HP, Kwan M, Amiel D, and Coutts RD. 1991. The use of polylactic acid matrix and periosteal grafts for the reconstruction of rabbit knee articular defects. *Journal of Biomedical Materials Research* 25:329-339. 10.1002/jbm.820250305
251 Wakitani S, Goto T, Pineda SJ, Young RG, Mansour JM, Caplan AI, and Goldberg VM. 1994. Mesenchymal cell-based repair of large, full-thickness defects of articular cartilage. *The Journal of Bone and Joint Surgery* 76:579-592.
252 Wang G, Li D, Gu GS, Sun DH, Wang CX, and Xu P. 2006. Influences of different inductive methods on cartilage repair by tissue engineered cartilage with rabbit mesenchymal stem cells. *Journal of Jilin University Medicine Edition* 32:981-984.
253 Wang H, and Wang W. 2014. Repairing articular cartilage defects using subchondral drilling and cartilage-derived morphogenetic protein delivery system. *Chinese Journal of Tissue Engineering Research* 18:3334-3340.
254 Wang W, Li B, Li Y, Jiang Y, Ouyang H, and Gao C. 2010. In vivo restoration of full-thickness cartilage defects by poly(lactide-co-glycolide) sponges filled with fibrin gel, bone marrow mesenchymal stem cells and DNA complexes. *Biomaterials* 31:5953-5965. 10.1016/j.biomaterials.2010.04.029
255 Wang W, Sun L, Zhang P, Song J, and Liu W. 2014a. An anti-inflammatory cell-free collagen/resveratrol scaffold for repairing osteochondral defects in rabbits. *Acta Biomaterialia* 10:4983-4995. 10.1016/j.actbio.2014.08.022
256 Wang X, Li Y, Han R, He C, Wang G, Wang J, Zheng J, Pei M, and Wei L. 2014b. Demineralized bone matrix combined bone marrow mesenchymal stem cells, bone morphogenetic protein-2 and transforming growth factor-beta3 gene promoted pig cartilage defect repair. *PLoS One* 9:e116061. 10.1371/journal.pone.0116061
257 Wang Y, Chen X, and Wang LG. 2007. Bone marrow monocytes and porous lactic acid mixture in the repair of rabbit articular cartilage defects. *Journal of Clinical Rehabilitative Tissue Engineering Research* 11:10128-10130.
258 Wayne JS, McDowell CL, Shields KJ, and Tuan RS. 2005. In vivo response of polylactic acid-alginate scaffolds and bone marrow-derived cells for cartilage tissue engineering. *Tissue Engineering* 11:953-963. 10.1089/ten.2005.11.953
259 Wegener B, Schrimpf FM, Bergschmidt P, Pietschmann MF, Utzschneider S, Milz S, Jansson V, and Muller PE. 2010. Cartilage regeneration by bone marrow cells-seeded scaffolds. *Journal of Biomedical Materials Research Part A* 95:735-740. 10.1002/jbm.a.32885
260 Wegener B, Schrimpf FM, Pietschmann MF, Milz S, Berger-Lohr M, Bergschmidt P, Jansson V, and Muller PE. 2009. Matrix-guided cartilage regeneration in chondral defects. *Biotechnology and Applied Biochemistry* 53:63-70. 10.1042/BA20080020
261 Wei B, Yao Q, Guo Y, Mao F, Liu S, Xu Y, and Wang L. 2015. Three-dimensional polycaprolactone-hydroxyapatite scaffolds combined with bone marrow cells for cartilage tissue engineering. *Journal of Biomaterials Applications*. 10.1177/0885328215575762

262 Wu J, Yang T, Liu Y, Guo T, Mu Y, and Li Y. 2008. Preliminary studies on repairing osteochondral defects in the rabbit knee joint by using porous PA66/n-HA combination mesenchymal stem cells. *Journal of Biomedical Engineering* 25:1349-1353.

263 Xie J, Han Z, Naito M, Maeyama A, Kim SH, Kim YH, and Matsuda T. 2010. Articular cartilage tissue engineering based on a mechano-active scaffold made of poly(L-lactide-co-epsilon-caprolactone): In vivo performance in adult rabbits. *Journal of Biomedical Materials Research Part B* 94:80-88. 10.1002/jbm.b.31627

264 Xie X, Wang Y, Zhao C, Guo S, Liu S, Jia W, Tuan RS, and Zhang C. 2012. Comparative evaluation of MSCs from bone marrow and adipose tissue seeded in PRP-derived scaffold for cartilage regeneration. *Biomaterials* 33:7008-7018. 10.1016/j.biomaterials.2012.06.058

265 Yagihashi K, Miyazawa K, Togari K, and Goto S. 2009. Demineralized dentin matrix acts as a scaffold for repair of articular cartilage defects. *Calcified Tissue International* 84:210-220. 10.1007/s00223-008-9205-7

266 Yan H, and Yu C. 2007. Repair of full-thickness cartilage defects with cells of different origin in a rabbit model. *Arthroscopy* 23:178-187. 10.1016/j.arthro.2006.09.005

267 Yan LP, Silva-Correia J, Oliveira MB, Vilela C, Pereira H, Sousa RA, Mano JF, Oliveira AL, Oliveira JM, and Reis RL. 2015. Bilayered silk/silk-nanoCaP scaffolds for osteochondral tissue engineering: In vitro and in vivo assessment of biological performance. *Acta Biomaterialia* 12:227-241. 10.1016/j.actbio.2014.10.021

268 Yang Q, Peng J, Lu SB, Guo QY, Zhao B, Zhang L, Wang AY, Xu WJ, Xia Q, Ma XL, Hu YC, and Xu BS. 2011. Evaluation of an extracellular matrix-derived acellular biphasic scaffold/cell construct in the repair of a large articular high-load-bearing osteochondral defect in a canine model. *Chinese Medical Journal* 124:3930-3938.

269 Yang YJ, and Zhu QS. 2009. Cartilage-derived morphogenetic protein growth factor 1-induced rat adipose-derived stem cells in repairing knee joint defect in rabbits. *Academic Journal of Second Military Medical University* 30:1363-1366. 10.3724/SP.J.1008.2009.01363

270 Yasuda K, Kitamura N, Gong JP, Arakaki K, Kwon HJ, Onodera S, Chen YM, Kurokawa T, Kanaya F, Ohmiya Y, and Osada Y. 2009. A novel double-network hydrogel induces spontaneous articular cartilage regeneration in vivo in a large osteochondral defect. *Macromolecular Bioscience* 9:307-316. 10.1002/mabi.200800223

271 Ye HZ, Li SM, Ye CT, Yang XH, Huang XF, and Liang PH. 2003. Animal experiment of type II collagen spongy used to repair articular cartilage defect. *Journal of Clinical Rehabilitative Tissue Engineering Research* 7:2405-2406.

272 Ye HZ, Li SM, Ye CT, Yang XH, Huang XF, and Liang PH. 2005. Repair of articular cartilage defect in rabbit with type II collagen sponge filling material. *Chinese Journal of Clinical Rehabilitation* 9:231-233.

273 Yin Z, Zhang L, and Wang J. 2005. Repair of articular cartilage defects with "two-phase" tissue engineered cartilage constructed by autologous marrow mesenchymal stem cells and "two-phase" allogeneic bone matrix gelatin. *Chinese Journal of Reparative and Reconstructive Surgery* 19:652-657.

274 Ylanen HO, Helminen T, Helminen A, Rantakokko J, Karlsson KH, and Aro HT. 1999. Porous bioactive glass matrix in reconstruction of articular osteochondral defects. *Annales Chirurgiae et Gynaecologiae* 88:237-245.

275 Yokota M, Yasuda K, Kitamura N, Arakaki K, Onodera S, Kurokawa T, and Gong JP. 2011. Spontaneous hyaline cartilage regeneration can be induced in an osteochondral defect created in the femoral condyle using a novel double-network hydrogel. *BMC Musculoskeletal Disorders* 12. 10.1186/1471-2474-12-49

276 Zha G, Liao W, Wang Y, and Wu G. 2008. Morphological observation on repairing articular cartilage defects with collagen complex gradient TCP. *Chinese Journal of Reparative and Reconstructive Surgery* 22:989-992.

277 Zhang N, and Wu Y. 2012. Thermosensitive chitosan-based hydrogel containing cytokines for rabbit full-thickness articular cartilage defects. *Chinese Journal of Tissue Engineering Research* 16:6298-6302.
278 Zhang S, Chen L, Jiang Y, Cai Y, Xu G, Tong T, Zhang W, Wang L, Ji J, Shi P, and Ouyang HW. 2013a. Bi-layer collagen/microporous electrospun nanofiber scaffold improves the osteochondral regeneration. *Acta Biomaterialia* 9:7236-7247. 10.1016/j.actbio.2013.04.003
279 Zhang W, Chen J, Tao J, Hu C, Chen L, Zhao H, Xu G, Heng BC, and Ouyang HW. 2013b. The promotion of osteochondral repair by combined intra-articular injection of parathyroid hormone-related protein and implantation of a bi-layer collagen-silk scaffold. *Biomaterials* 34:6046-6057. 10.1016/j.biomaterials.2013.04.055
280 Zhang W, Lian Q, Li D, Wang K, Hao D, Bian W, He J, and Jin Z. 2014. Cartilage repair and subchondral bone migration using 3D printing osteochondral composites: a one-year-period study in rabbit trochlea. *BioMed Research International* 2014:746138. 10.1155/2014/746138
281 Zhao Q, Wang S, Tian J, Wang L, Dong S, Xia T, and Wu Z. 2013. Combination of bone marrow concentrate and PGA scaffolds enhance bone marrow stimulation in rabbit articular cartilage repair. *Journal of Materials Science: Materials in Medicine* 24:793-801. 10.1007/s10856-012-4841-x
282 Zhao R, Peng X, Chu H, and Song W. 2014. Phosphorylatable short peptide conjugated chitosan mediated gene therapy for repair of articular cartilage defect in rabbits. *Chinese Journal of Reparative and Reconstructive Surgery* 28:1346-1352.
283 Zhou XZ, Leung VY, Dong QR, Cheung KM, Chan D, and Lu WW. 2008. Mesenchymal stem cell-based repair of articular cartilage with polyglycolic acid-hydroxyapatite biphasic scaffold. *The International Journal of Artificial Organs* 31:480-489.
