# Supplementary figures and images for: Improved cartilage regeneration by implantation of acellular biomaterials after bone marrow stimulation: a systematic review and meta-analysis of animal studies
Source: PeerJ. 2016 Sep 8;4:e2243. doi: 10.7717/peerj.2243 (PMC5018675; doi:10.7717/peerj.2243)

Supplementary Information 7. Forest plot.

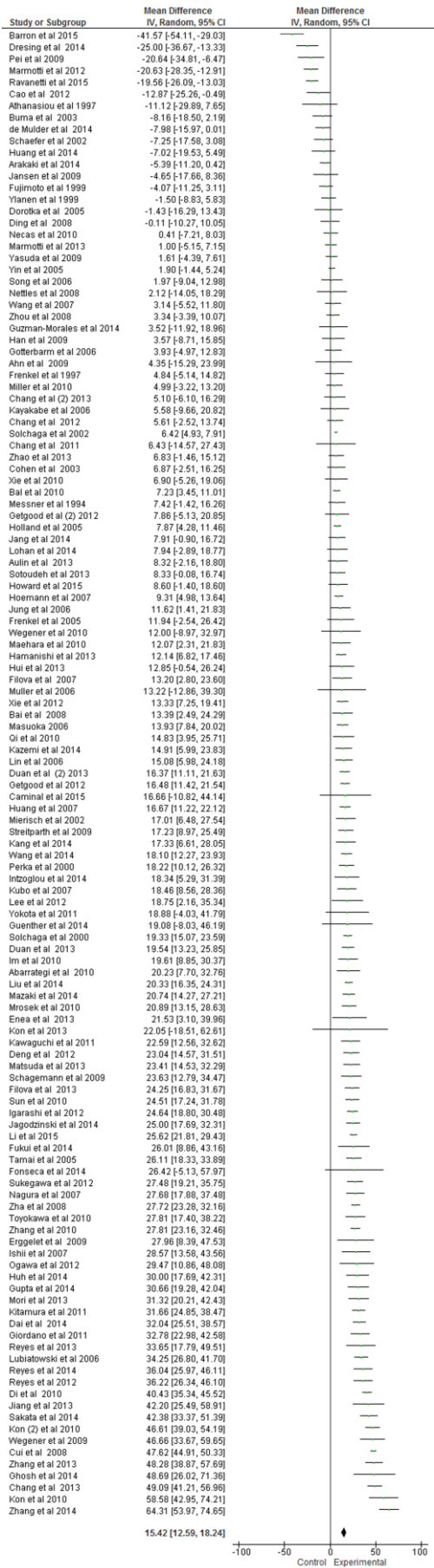

Supplement: Supplemental Information 7 [file peerj-04-2243-s007.pdf]
